# Immunomodulatory and anticancer effects of moringa polyherbal infusions: potentials for preventive and therapeutic use

**DOI:** 10.3389/fimmu.2025.1597602

**Published:** 2025-06-19

**Authors:** Wamidh H. Talib, Hadeel Shaher Al Junaidi, Heba K. Alshaeri, Moudi M. Alasmari, Rawan W. Hadi, Ahmad Riyad Alsayed, Douglas Law

**Affiliations:** ^1^ Faculty of Allied Medical Sciences, Applied Science Private University, Amman, Jordan; ^2^ Faculty of Health and Life Sciences, Inti International University, Nilai, Malaysia; ^3^ Department of Clinical Pharmacy and Therapeutics, School of Pharmacy, Faculty of Pharmacy, Applied Science Private University, Amman, Jordan; ^4^ Department of Pharmacology, Faculty of Medicine, King Abdul-Aziz University, Rabigh, Saudi Arabia; ^5^ College of Medicine, King Saud bin Abdulaziz University for Health Sciences (KSAU-HS), Jeddah, Saudi Arabia; ^6^ King Abdullah International Medical Research Centre (KAIMRC), Jeddah, Saudi Arabia; ^7^ Department of Clinical Pharmacy and Therapeutics, Applied Science Private University, Amman, Jordan

**Keywords:** herbal infusions, green tea, immunomodulatory, anticancer, herbal supplements

## Abstract

**Introduction:**

Plant-based phytochemicals have shown potential as agents against cancer through various biological pathways, including immune system modulation. Combining natural compounds with synergistic interactions enhances the identification of molecular targets in cancer cells, demonstrating anticancer capabilities. The “miracle tree,” *Moringa oleifera* (Moringa), possesses remarkable nutritional and medicinal properties.

**Aim:**

This study assesses the anti-tumor and immunomodulatory characteristics of *M. oleifera* aqueous leaf extract and its combinations with green tea, saffron, lavender, and turmeric.

**Methods:**

The MTT assay evaluated the anti-proliferative effectiveness of uncombined *M. oleifera* and its combinations against three breast cancer cell lines. Quantitative LC-MS/MS spectrometry was performed on the most potent extracts. The Nitro Blue Tetrazolium Assay and Neutral Red Method were used to assess the effects of each extract on macrophage phagocytic and pinocytosis functions. Lymphocyte proliferation was also tested.

**Results:**

The anti-proliferative activity of Moringa-saffron showed the strongest anti-proliferative effect on T47-D and EMT6/P, with IC_50_ values of 1.14 ± 0.05 and 1.65 ± 0.27 mg/ml, respectively. The Moringa-green tea combination exhibited the highest anti-proliferative action against MDA-MB-231 among all combinations, with a 5.78 ± 0.06 mg/ml IC_50_ value. Moringa-lavender and Moringa-turmeric extracts showed minimal antiproliferation action. In contrast, uncombined Moringa had the lowest activity on the tested cell lines, with an IC_50_ greater than 6 mg/ml. For the immunomodulatory tests, Moringa-saffron and Moringa-green tea were the most effective combinations; they significantly induced macrophage phagocytosis, pinocytosis, and lymphocyte proliferation simultaneously. The LC-MS/MS analysis revealed the presence of flavonoids, phenols, coumarins, and alkaloids in the Moringa-saffron and Moringa-green tea combinations, which, along with their immune modulation activities, could contribute to their anticancer potential.

**Conclusion:**

When combined with saffron or green tea, Moringa can effectively combat cancer and enhance immunomodulatory properties. Further studies are needed to understand the mechanism of action of these herbal infusions.

## 1 Introduction

Cancer, often referred to as “the sickness of the century,” remains one of the leading causes of death worldwide. Its increasing incidence and resistance to existing pharmacological treatments pose significant clinical challenges. According to the American Cancer Society, over 1.8 million new cases and more than 600,000 cancer-related deaths occurred in the United States in 2020 alone (1). Global projections estimate 17 million cancer deaths and 26 million new cases annually by 2030 (2), highlighting the urgent need for novel and more effective chemotherapeutic and chemopreventive agents (3).

Current treatment options include chemotherapy, surgery, and radiation. However, they are restricted by toxicity, resistance, and incomplete efficacy (4, 5).

Extensive research has assessed the efficacy of various natural chemicals and their potential use in medicinal applications. The therapeutic potential of phytochemicals from medicinal plants has been recognized for treating numerous ailments. Many researchers are documenting the biological activities of these plant-derived compounds against key pathways in disease progression, thus strengthening support for their roles as natural therapeutic agents (6–8).

Natural products have played a crucial role in modern drug development, particularly cancer therapy. Between 1981 and 2002, approximately 62% of anticancer drugs approved in the United States were derived from natural sources (9).

Plant-derived compounds are fascinating due to their multitarget mechanisms and minimal side effects—many exhibit immunomodulatory, antioxidant, and anti-proliferative activities (10, 11). Immunotherapy has recently revolutionized cancer treatment, with 17 immunologic products approved for clinical use over the past 25 years (12). Natural products with immunomodulatory potential are promising for future cancer therapies (13).

*Moringa oleifera* (MO), known as the Miracle Tree, has gained attention for its high nutritional value and extensive medicinal properties. Native to the Indian subcontinent and now widely distributed in tropical regions, MO has been used traditionally for over 5,000 years (14–16).

It exhibits hepatoprotective, antimicrobial, diuretic, antiulcer, and antioxidant activities. Its bioactive components—phenolics, flavonoids, isothiocyanates, and glucosinolates—contribute to its anticancer, antiangiogenic, and immunomodulatory effects (17).

In the U.S., up to 60% of cancer patients use dietary supplements or plant-based remedies alongside conventional therapies (18, 19). Numerous natural compounds, such as sulforaphane (broccoli), genistein (soy), curcumin (turmeric), EGCG (green tea), resveratrol (grapes), and silymarin (milk thistle), have shown promise in cancer prevention or treatment (20). Saffron and lavender also display notable anticancer and immunomodulatory activities (21–24). Saffron has been shown to induce apoptosis in MCF-7 breast cancer cells via caspase activation (25, 26), while lavender has demonstrated efficacy against multiple cancer cell lines and in xenograft tumor models (27, 28).

Similarly, green tea consumption has reduced breast cancer risk and improved prognosis in early-stage disease (29, 30). Turmeric’s active compound, curcumin, modulates various cancer-related pathways, including DNA methylation, and exhibits strong protective effects against breast cancer (31, 32).

This study examined the anti-proliferative and immune system activation properties of Moringa aqueous leaf extract and its combinations with lavender, turmeric, saffron, and green tea. These combinations were selected particularly because they are widely consumed by the local population for their health benefits and general well-being. However, research on their combined anticancer and immunomodulatory effects is insufficient, while separate drugs demonstrate these effects. Furthermore, a phytochemical analysis of the most effective combinations was conducted to elucidate the phytochemicals present.

## 2 Materials and methods

### 2.1 Reagents, instruments, and commercial kits

In the current study, the following equipment, supplies, and kits were used: a 96-well cell culture plate (Biofil, Guangzhou, China); a UV spectrophotometer (Lab-Line Instruments Incorporation, Kerper Blvd, Dubuque, IA, USA); a refrigerated centrifuge (Denley, UK); a hemocytometer (Neubauer, Erzhausen, Germany); a Universal Extractor apparatus (Buchi, mod. E-800, Uster, Switzerland); a lyophilizer (Edwards, Burgess Hill, UK); a light microscope (Meiji Techno Co. LTD, Japan); and an ELISA microplate absorbance reader (BioTek, Highland Park, IL, USA). The following supplies were provided by Sigma, St. Louis, MO, USA: trypan blue 0.4%, DMEM (Dulbecco’s modified Eagle medium), MEM (minimum essential medium), FBS (fetal bovine serum), L-glutamine, penicillin-streptomycin solution, gentamicin, PBS (phosphate-buffered saline), and trypsin–EDTA (ethylenediamine tetraacetic acid). An MTT (3-(4, 5-dimethylthiazol-2-yl)-2, 5-diphenyltetrazolium bromide) kit (Bioworld, London, UK) was used to carry out the anti-proliferative experiment.

### 2.2 *Moringa oleifera* collection and extraction using the maceration method

The maceration extraction method was chosen because it resembles the traditional preparation and consumption of herbal infusions in our area, where the herbs are typically steeped in hot water before consumption. Our goal was to accurately replicate this authentic practice to reflect the conditions under which these infusions are prepared and consumed.

The following quantities of dried *M. oleifera* (Moringa) leaves and various ready Moringa herbal combinations were sourced from MORINGA JORDAN. Four hundred milliliters of distilled water were gently boiled with 12 grams of dried leaves, 16 grams of Moringa-green tea combination, 18 grams of Moringa-turmeric combination, 12 grams of Moringa-lavender combination, and 12 grams of Moringa-saffron combination for the extraction process. The extracted materials were covered and left to fully extract overnight by soaking. After this, the extracts were filtered and evaporated using a rotary evaporator to a dry state, followed by lyophilization for two days to completely dry the aqueous extracts, which were then stored under refrigeration until examination.

### 2.3 Cell culturing and conditions

MDA-MB-231, EMT6/P, and T47-D were the three cell lines selected to study the potential anti-proliferative effects of *M. oleifera* extracts. The Vero cell line, originating from the American Type Culture Collection (ATCC, Manassas, VA, USA), was used to assess the impact of *M. oleifera e*xtracts on normal cells. Additional components included in the tissue culture media were 10% fetal bovine serum, 1% penicillin-streptomycin solution, 1% L-glutamine, 0.1% non-essential amino acid solution, and 0.1% gentamicin solution. The following volumetric ratios of the components were added to the media (as specified) in 500 mL bottles: 50 mL, 5 mL, 5 mL, 0.5 mL, and 0.5 mL, respectively. The T47-D cell line was cultured in RPMI 1640, while the Vero and EMT6/P cell lines were grown in MEM. The MDA-MB-231 cell line was cultivated in complete DMEM high-glucose media, and the cells were grown in the incubator until they reached 80-90% confluence.

### 2.4 Phytochemical analysis of *M. oleifera* and its combinations using LC-MS/MS

The *M. oleifera* and its four combinations were dissolved in distilled water to create freshly prepared aqueous samples. These samples were filtered using a 0.45 μm membrane filter (cellulose acetate (CA) membranes, made entirely of cellulose acetate polymer by Sterlitech, Auburn, WA, USA). Each sample was analyzed immediately after preparation. To evaluate the extracts using the Exion LC technique, an X500 QTOF mass spectrometer (SCIEX, Framingham, MA, USA) connected to an ESI was utilized. Separation was performed using an InertSustain C18 column (GL Sciences Inc., Tokyo, Japan, 25 cm × 4.6 mm × 5 μm). The mobile phase consisted of two solutions: (A) acetonitrile and (B) 0.1% formic acid in water (1:1000), with a gradient flow rate of 1.0 ml/min. A volume of 0.6 μL was reserved for injection. The LC-MS/MS was operated in positive mode with an ion spray voltage of 5000 V, a collision energy of 10 V, and a declustering potential of 80 V. The NIST (National Institute of Standards and Technology, USA) mass spectrum library was used for material identification.

### 2.5 Anti-proliferative assay

The MTT assay (3-(4,5-dimethylthiazol-2-yl)-2,5-diphenyltetrazolium bromide) was employed to assess the anti-proliferative effectiveness of the extracts. The cells were titrated to 1.5 × 10^4 cells per ml in each well of a 96-well cell culture plate. These cells were left to incubate for 5 days at 37°C in an environment of 5% CO_2_ and 95% humidity. Subsequently, varying quantities of *M. oleifera* extract (0.04–6 mg/ml) were added. Using the MTT test kit, the viability of the cells was evaluated over a 48-hour incubation period. This was accomplished by adding 10 µl of thiazolyl blue tetrazolium solution to each well and then incubating the plate for three hours. The addition of DMSO dissolves the formazan particles that developed in the viable cells. A microplate reader was used to measure the optical density (OD) at 550 nm after incubating the plate for an hour. The IC50 values and the percentage of surviving cells were computed using SPSS 25.


$$cell\;viability\%\;=\frac{optical\;density\;of\;treated\;cells}{optical\;density\;of\;control\;cells\;}*100$$

### 2.6 Animals

Balb/C mice were used in the immune modulation study, weighing between 23 and 25 grams, with an average age of 4 to 6 weeks. The animal chamber was maintained at 25°C with continuous air ventilation and 50 to 60% humidity. They were provided food, water, and sterile nourishment on a 12-hour light/dark cycle in a pathogen-free environment. The Institutional Review Board (IRB) in the Faculty of Pharmacy, Applied Science Private University, approved all the experimental protocols used in this research (Approval Number: 2024-PHA-50) that were conducted following accepted ethical standards.

### 2.7 Immune assays

#### 2.7.1 Murine splenocytes preparation

To produce murine splenocytes, a slaughtered Balb/C mouse was euthanized, and its spleen was aseptically removed. After the spleen was ground through the mesh of a tissue grinder, a cell suspension was created, and the mixture was added to RPMI-1640 medium. After being transferred to a centrifuge tube, the cell mixture was centrifuged at 1500 RPM and 4°C for ten minutes to extract the red blood cells, discarding the supernatant and resuspending the cells in RBC cell lysis buffer. Before centrifugation, the suspension needed to be pipetted several times. The splenocytes were then ready for seeding and counting in different assays after the pellets were resuspended in 5 milliliters of RPMI-1640 medium.

#### 2.7.2 Lymphocyte proliferation assay

This assay used the MTT (3-(4, 5-dimethylthiazol-2-yl)-2, 5-diphenyltetrazolium bromide) test kit. A 96-well tissue culture plate was seeded with a suspension of splenocytes at a known concentration of 2 × 10^6^ cells/ml. Then, 100 μl of *M. oleifera* and the combination extracts were added; the experiment was conducted in triplicate with different concentrations of 0.75, 1.5, 3, and 6 mg/ml. The plate was incubated at 37°C in a humidified atmosphere with 5% CO_2_ for 48 hours. Next, ten microliters of MTT were added to each well and incubated for four hours. After dissolving the formazan particles with 100 μL of dimethyl sulfoxide solution (DMSO), the absorbance was measured using the microplate reader ELISA at 550 nm. The results were represented as stimulation index values (33).


$$stimulation\;index=\frac{OD\;of\;stimulated\;cells\;\left(treated\right)}{\;OD\;of\;unstimulated\;cells\;\left(control\right)}$$

#### 2.7.3 Isolation of murine macrophages

Due to its high density, the peritoneal cavity is a preferred site for accumulating macrophages. To enhance the yield of macrophages, the mice were first administered an injection of 3 ml of 3% (w/v) brewer’s thioglycollate medium into the peritoneal cavity, following the procedure described by Ray and Dittel (34). After initiating macrophage development over three to five days, the next step involved cell collection. The peritoneal macrophages (PEM) were separated using sterile, ice-cold phosphate-buffered saline (pH 7.4). Following cervical dislocation, the mice were sacrificed, and their abdominal cavities were opened to facilitate the injection of 5 milliliters of ice-cold PBS. The peritoneum was gently rubbed to release any adhered cells into the PBS solution. The fluid was carefully removed, placed in a centrifuge tube, and repeated multiple times to obtain the required volume. The cell pellets were collected by centrifugation at 1500 RPM and 4°C for 10 minutes and resuspended in RPMI 1640 media afterward.

#### 2.7.4 Phagocytic activity of macrophages using the NBT (nitro blue tetrazolium) reduction method

The technique described in Boothapandi and Ramanibai was used to complete the NBT reduction assay (34). In summary, 96-well tissue culture plates were seeded with peritoneal macrophages at a density of 5 × 10^6^ cells/well. Except for the control wells, they were treated with various doses of *M. oleifera* extracts (0.75, 1.5, 3, and 6 mg/ml) and incubated for 48 hours at 37°C. Afterwards, each well received 20μL of nitro blue tetrazolium (NBT) (1.5 mg/ml in PBS) and 20μL of yeast suspension (5 × 10^7^ cells/ml in PBS). The plate was incubated at 37°C for sixty minutes. After the incubation period, RPMI 1640 was used to rinse the adherent macrophage cells, drain the supernatant, and allow them to air dry. Next, 120 μl of 2M KOH and 140 μl of DMSO were added to each well. Then, the plate was placed on a microplate reader, and the optical density (OD) was measured at 550 nm. The following formula was used to calculate the phagocytic activity, which was reported as the percentage of NBT reduction.


$$phagocytic\;index=\frac{\;sample\;OD\;-\;Control\;OD\;}{control\;OD\;}*100$$

#### 2.7.5 Pinocytic activity of macrophages using the neutral red method

The mechanism in question was expounded upon by Boothapandi and Ramanibai (34). To summarize, peritoneal macrophages were seeded at a concentration of 5×10^6^ cells per well in a 96-well tissue culture plate. Except for the control wells, various amounts of *M. oleifera* extracts (0.75, 1.5, 3, and 6 mg/ml) were added, and the plate was incubated for 48 hours at 37°C. Next, 100 μl of neutral red solution (7.5 mg/ml in PBS) was added to each well, and the mixture was incubated for two hours. Following the incubation period, the supernatant was discarded, and each well underwent two rinses with PBS to remove any neutral red that the macrophages had not pinocytized. Subsequently, 100 μl of the cell lysis solution (ethanol and 0.01% acetic acid at a 1:1 ratio) was added to each well to initiate the breakdown of the cells. The dish was left at room temperature overnight. The optical density (OD) at 550 nm was measured after the plate was placed on a microplate reader. The absolute optical density values depicted the dye uptake, signifying the presence of pinocytic activity.

### 2.8 Statistical analysis

To determine whether there were significant differences in the mean values for each parameter among the various groups in the experiment, one-way ANOVA and *post hoc* analysis using Dunnett’s multiple comparisons test were applied to represent the data in SPSS (Statistical Package for the Social Science, Chicago, IL, USA, version 24). All assays were performed in technical triplicate (n=3), and the entire experiment was repeated independently three times (biological replicates, N=3). Data are presented as mean ± SEM. When p < 0.05, the mean values of the groups were considered significantly different. Nonlinear regression in SPSS was employed to obtain IC_50_ values.

## 3 Results

### 3.1 yield% of crude extract

After the extraction process, the percentage yield of each of the five aqueous extracts varied. The highest yield came from the Moringa-lavender combination, followed by the Moringa-saffron, uncombined Moringa, Moringa-green tea, and Moringa-turmeric combinations, respectively. The percent yield for each solvent extract was calculated using the formula provided below (**Table 1**).

**Table 1:** Percentage yield of the Moringa leaves aqueous extract and the four different Moringa combination extracts.

Extract	Weight before extraction (g)	Weight after extraction (g)	% yield of dried leaf aqueous extracts
Moringa	12	1.59	13.25%
Moringa- green tea	12	1.34	11.16%
Moringa-lavender	10	1.9	19%
Moringa-saffron	12	1.89	15.75%
Moringa- turmeric	18	0.83	4.61%


$$percent\;of\;obtained\;yeild\;\left(\%yeild\right)=\frac{Actual\;yeild}{theoritical\;yeild\;}*100$$

### 3.2 Quantitative analysis of Moringa green tea and Moringa saffron extracts using LC-MS/MS

After it was determined that the combinations of Moringa-green tea and Moringa-saffron had the most excellent anti-proliferative effect, LC-MS/MS analysis was carried out on these combinations’ extracts, revealing the presence of several bioactive phytochemicals. Results showed that Moringa-saffron leaf extract contained phenolic compounds such as Demethoxycurcumin and 4-O-Caffeoylquinic acid. This combination also possessed a fatty acid (11,12-Dihydroxy-5Z,8Z,14Z-eicosatrienoic acid), a steroidal lactone (Withanone), a coumarin (6,7-Dimethoxy-4-ethylcoumarin), flavonoids such as Iridin, Pinocembrin, Luteolin-7,3’-di-O-glucoside, and 3-Formyl-6-isopropylchromone, and included the alkaloid Harmol.

However, the Moringa-green tea combination contained phenols (beta-D-glucosiduronic acid, Demethoxycurcumin, 4,4’-Dimethoxystilbene, (-)-Gallocatechin 3-gallate, and (-)-Quinic acid). It also included flavonoids (Iridin, 6,2’-Dimethoxy-3-hydroxyflavone, Flavanomarein, and Quercetagetin-7-O-glucoside) and the coumarin dimethylfraxetin. The Moringa-green tea combination was rich in alkaloids such as harmol, glaucine, and huperzine. Other nitrogen-containing compounds, N-desethylsunitinib and pheophorbide A, were unique to this combination. In contrast to the Moringa and saffron combination, this blend also contains the benzoquinone thymoquinone. **Table 2** lists the phytochemicals found in each combination and the relative percentage for each compound.

**Table 2:** LC-MS/MS analysis for aqueous extracts of Moringa-green tea and Moringa-saffron combinations.

No.	Compound	Formula	m/z*	Rt	Relative percent (MO-green tea)	Relative percent (MO-saffron)
1	beta.-D-Glucopyranosiduronic acid, 4-3-(1-naphthalenylcarbonyl)-1H-indol-1-ylbutyl	C_21_H_18_O_11_	519.3353	24.23	11.4475	ND*
2	11,12-Dihydroxy-5Z,8Z,14Z-eicosatrienoic acid	C_20_H_34_O_4_	338.13959	9.55	ND	15.41856
3	Demethoxycurcumin	C_20_H_18_O_5_	338.13924	10.93	5.212386	4.354124
4	Withanone	C_28_H_38_O_6_	470.08423	17.34	ND	2.93517
5	4-O-Caffeoylquinic acid	C_16_H_18_O_9_	354.13648	10.2	ND	1.802297
6	Ureidosuccinic acid	C_5_H_8_N_2_O_5_	320.25285	23.35	ND	4.20529
7	5-Hydroxyisovanillic acid	C_8_H_8_O_5_	184.06219	10.32	ND	1.56951
8	6,7-Dimethoxy-4-ethylcoumarin	C_13_H_14_O_4_	234.08046	14.24	ND	0.36983
9	Iridin	C_24_H_26_O_13_	522.26733	12.77	0.150697	0.369385
10	Pinocembrin	C_15_H_12_O_4_	256.10530	20.05	ND	0.538121
11	Luteolin-7,3’-di-O-glucoside	C_27_H_30_O_16_	610.22020	11.74	ND	0.496426
12	3-Formyl-6-isopropylchromone	C_13_H_12_O_3_	216.06842	11.01	ND	0.67455
13	(-)-Gallocatechin 3-gallate	C_22_H_18_O_11_	458.13651	11.27	2.635173	ND
14	6,2’-Dimethoxy-3-hydroxyflavone	C_17_H_14_O_5_	298.19634	20.84	2.826577	ND
15	Dimethylfraxetin	C_12_H_12_O_5_	236.14084	16.76	4.60513	ND
16	N-Desethylsunitinib	C_20_H_23_FN_4_O_2_	370.09665	6.2	4.89585	ND
17	4,4’-Dimethoxystilbene	C_16_H_16_O_2_	240.06357	12.28	0.74575	ND
18	Harmol	C_12_H_10_N_2_O	198.12533	21.81	0.68972	0.28818
19	Glaucine	C_21_H_25_NO_4_	355.06599	4.5	0.55236	ND
20	Pheophorbide a	C_35_H_36_N_4_O_5_	592.21739	13.29	0.52498	ND
21	Flavanomarein	C_21_H_22_O_11_	450.16783	11.06	0.37782	ND
22	Quercetagetin-7-O-glucoside	C_21_H_20_O_13_	498.15626	14.12	0.281579	ND
23	Thymoquinone	C_10_H_12_O_2_	132.05841	7.12	0.34372	ND
24	Huperzine A	C_15_H_18_N_2_O	242.08594	27.92	0.43439	ND
25	(-)-Quinic acid	C_7_H_12_O_6_	192.06375	2.92	0.89785	ND

*ND, not detected; Rt, retention time; m/z, mass-to-charge ratio.

### 3.3 Activity of *M. oleifera* and its combination extracts on the proliferation of cancer cell lines

**Figure 1:**
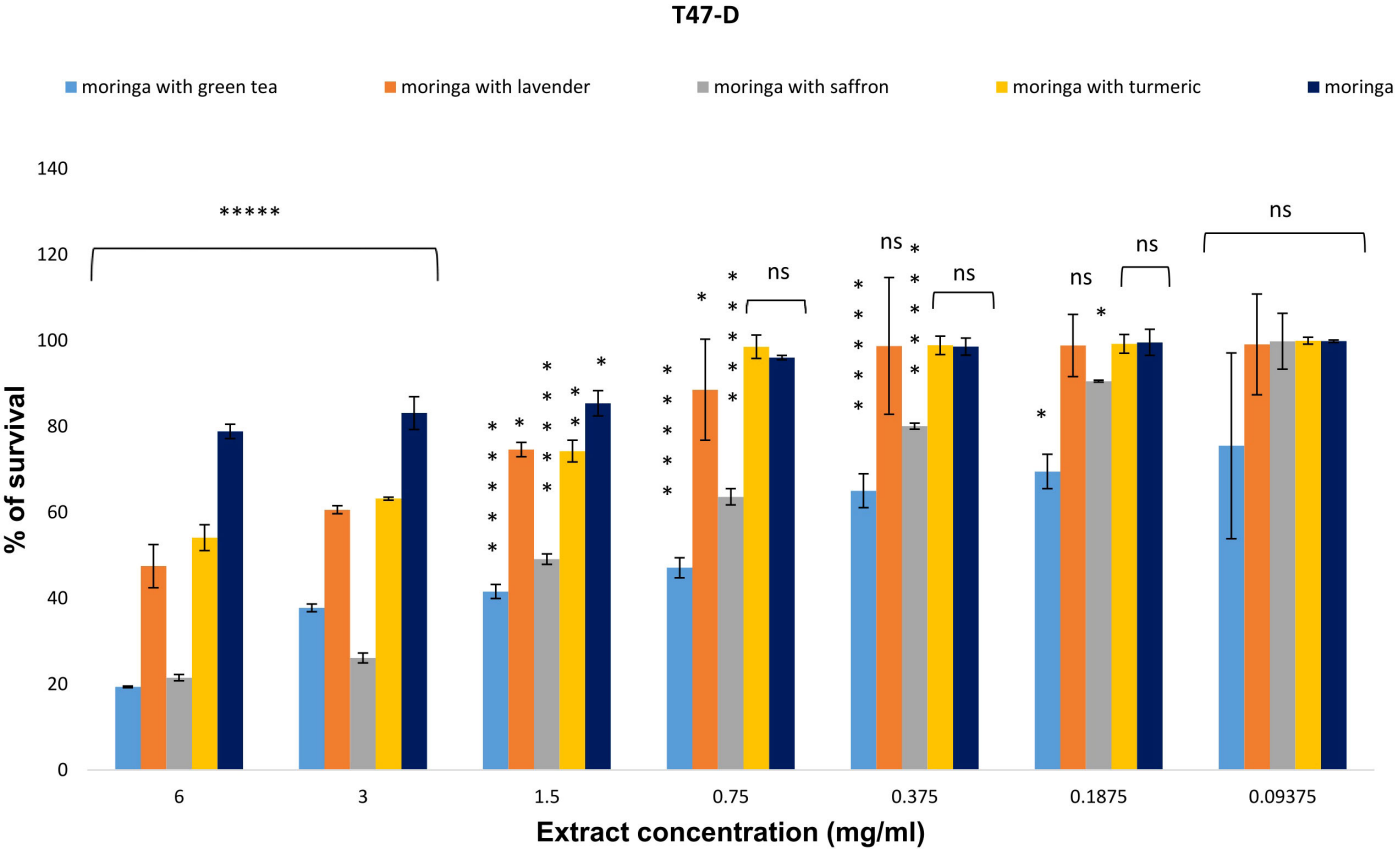
The anti-proliferative potential of *Moringa oleifera* aqueous extract and its combinations was evaluated against the T47-D cell line. Results are represented as mean ± SEM. Statistical comparisons were made against the negative control group (n = 3 independent experiments in technical triplicate). Statistical analysis was conducted using one-way ANOVA and Dunnett’s *post hoc* test. Significance is indicated as (*****) *p*-value < 0.000001, (**) *p*-value < 0.01, (*) *p*-value < 0.05, and (ns) for non-significant differences. Treatment concentrations were compared with the mean of the negative control group. A *p*-value < 0.05 was considered statistically significant. Error bars represent the SEM.

For all cell lines, the average survival percentage increased in a dose-dependent manner as the concentration of Moringa (*M. oleifera*) and its combined extracts was progressively reduced (0.09375–6 mg/ml). In conclusion, the survival percentages for the combinations of Moringa with green tea, lavender, saffron, turmeric, and uncombined Moringa, at a concentration of 6 mg/ml on the T47-D cell line, were 19.357%, 47.488%, 21.519%, 54.132%, and 78.862%, respectively. The Moringa-saffron combination was the most effective extract, followed by Moringa-green tea and Moringa-lavender, with an IC_50_ of 1.14 ± 0.05, 1.49 ± 0.1, and 5.9 ± 2.4, respectively, while uncombined Moringa and Moringa-turmeric were the least active, having an IC_50_ greater than 6 mg/ml (**Figure 1**).

**Figure 2:**
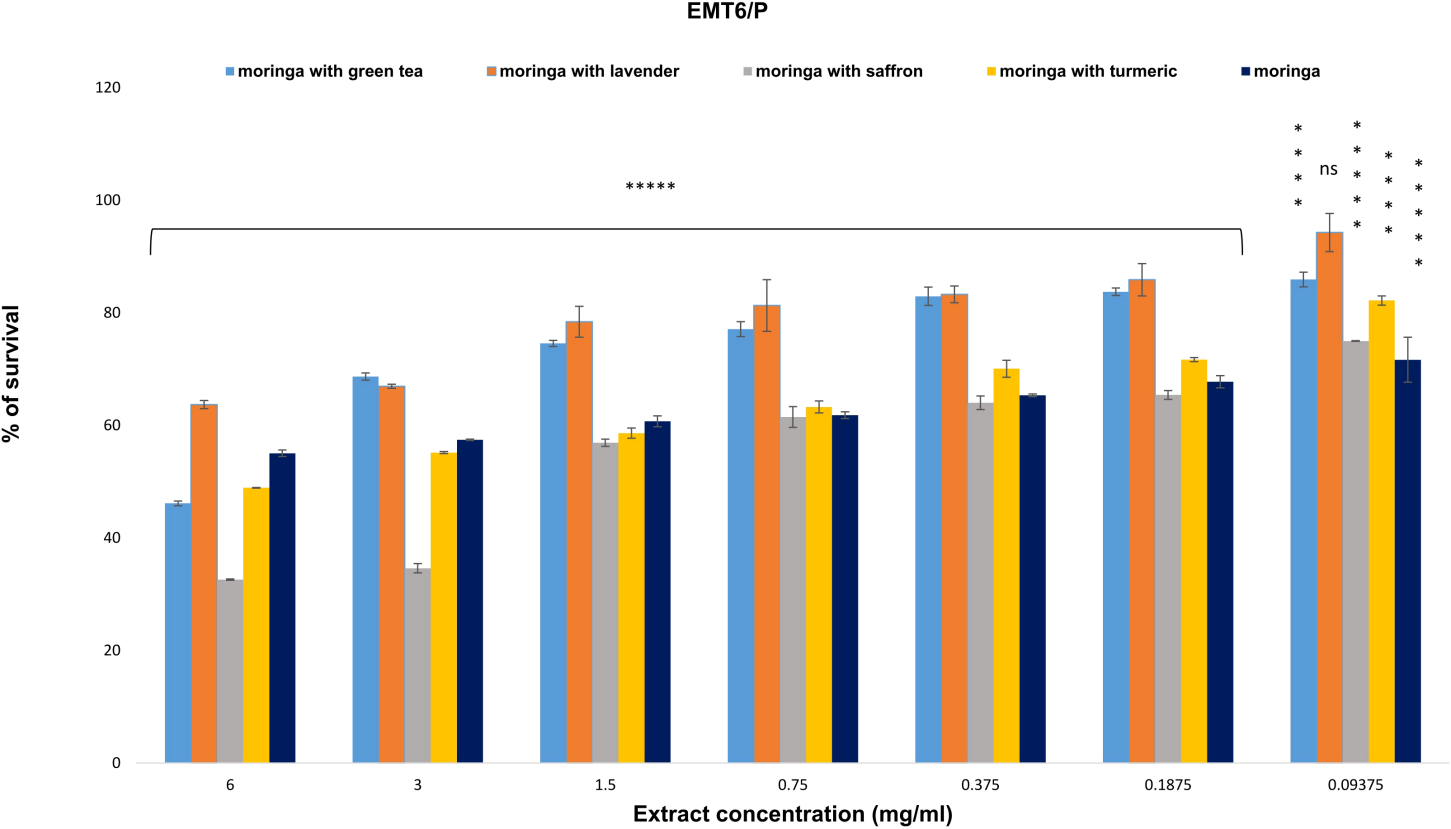
The anti-proliferative potential of *Moringa oleifera* aqueous extract and its combinations was evaluated against the EMT6/P cell line. Results are represented as mean ± SEM. Statistical comparisons were made against the negative control group (n = 3 independent experiments in technical triplicate). Statistical analysis was conducted using one-way ANOVA and Dunnett’s *post hoc* test. Significance is indicated as (*****) *p*-value < 0.000001, (****) *p*-value < 0.0001, and (ns) for non-significant differences. Treatment concentrations were compared with the mean of the negative control group. A *p*-value < 0.05 was considered statistically significant. Error bars represent the SEM.

On the EMT6/P cell line, the survival percentages for the combinations of Moringa with green tea, lavender, saffron, turmeric, and uncombined Moringa at a concentration of 6 mg/ml were 46.140%, 63.705%, 32.588%, 48.922%, and 55.042%, respectively. The highest anti-proliferative activity was observed in the combinations of Moringa with saffron, green tea, and turmeric, with IC_50_ values of 1.65 ± 0.27, 5.78 ± 0.06, and 5.93 ± 0.21, respectively. Conversely, the uncombined Moringa and the Moringa-lavender combination exhibited the least anti-proliferative activities, with IC_50_ values higher than 6 mg/ml (**Figure 2**).

**Figure 3:**
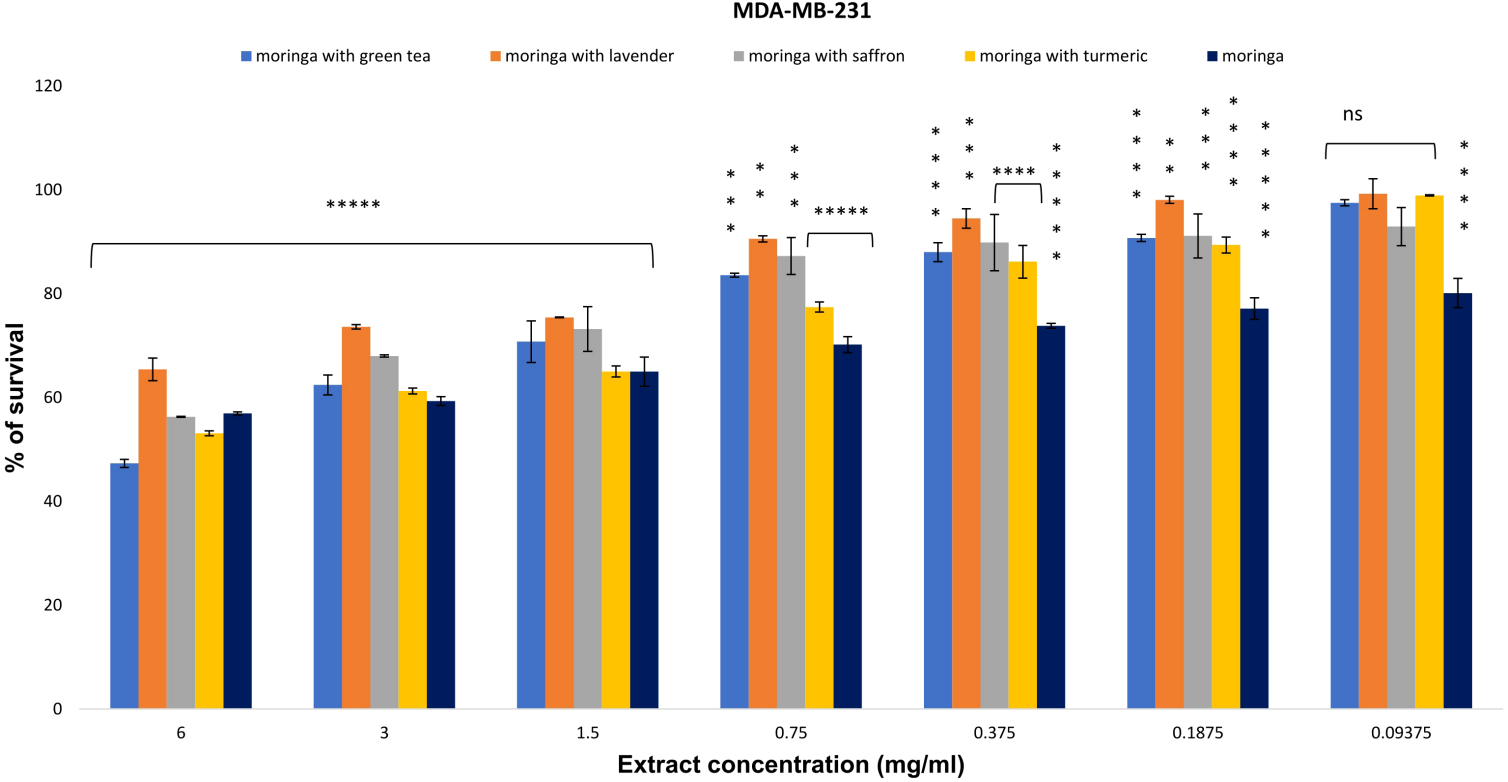
The anti-proliferative potential of *Moringa oleifera* aqueous extract and its combinations was evaluated against the MDA-MB-231 cell line. Results are represented as mean ± SEM. Statistical comparisons were made against the negative control group (n = 3 independent experiments in technical triplicate). Statistical analysis was conducted using one-way ANOVA and Dunnett’s *post hoc* test. Significance is indicated as (*****) *p*-value < 0.000001, (****) *p*-value < 0.0001, (***) *p*-value < 0.001, (**) *p*-value < 0.01, and (ns) for non-significant differences. Treatment concentrations were compared with the mean of the negative control group. A *p*-value < 0.05 was considered statistically significant. Error bars represent the SEM.

For MDA-MB-231, the survival percentages at a 6 mg/ml extract concentration for the combinations of Moringa-green tea, Moringa-turmeric, Moringa-saffron, Moringa-lavender, and uncombined Moringa were 47.323%, 65.451%, 56.299%, 53.106%, and 56.937%, respectively. The MDA-MB-231 cell line showed significant resistance to Moringa and its combinations, with an IC_50_ greater than 6 mg/ml, except for the combination of Moringa and green tea, which exhibited the highest activity with an IC_50_ of 5.7 ± 0.2 mg/ml (**Figure 3**).

**Figure 4:**
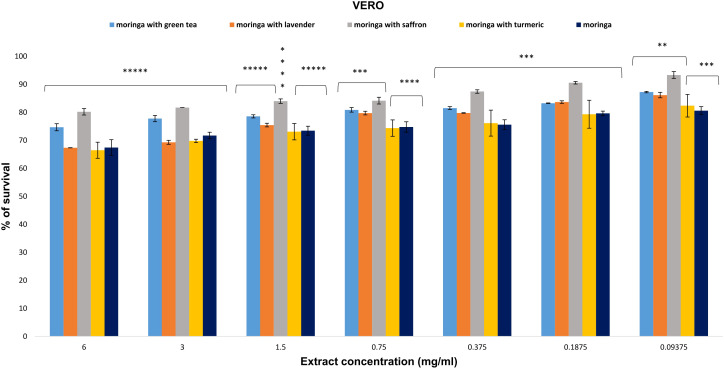
The anti-proliferative potential of *Moringa oleifera* aqueous extract and its combinations was evaluated against the VERO cell line. Results are represented as mean ± SEM. Statistical comparisons were made against the negative control group (n = 3 independent experiments in technical triplicate). Statistical analysis was conducted using one-way ANOVA and Dunnett’s *post hoc* test. Significance is indicated as (*****) *p*-value < 0.000001, (****) *p*-value < 0.0001, (***) *p*-value < 0.001, and (**) *p*-value < 0.01 significance level. Treatment concentrations were compared with the mean of the negative control group. A *p*-value < 0.05 was considered statistically significant. Error bars represent the SEM.

The VERO cell line showed greater resistance to the studied Moringa extracts, as indicated by the proportion of living cells. All extracts exhibited limited toxicity on normal cells, with an IC_50_ value exceeding 6 mg/ml (**Figure 4**).

### 3.4 The IC_50_ values of uncombined Moringa and its combinations’ aqueous extracts tested against MDA-MB-231, EMT6/P, T47-D, and Vero normal cell lines

The term IC_50_ refers to the concentration of a material needed to kill 50% of cells compared to the negative control. The IC_50_ (mg/ml) values for the crude extracts of uncombined Moringa and its various combinations were assessed on four cell lines (**Table 3**).

The combination of Moringa and saffron exhibited the highest anti-proliferative activity on T47-D and EMT6/P, with IC_50_ values of 1.14 ± 0.05 and 1.65 ± 0.27 mg/ml, respectively. Meanwhile, it showed the lowest activity on MDA-MB-231, with an IC_50_ greater than 6 mg/ml.

The Moringa-green tea combination exhibited the highest anti-proliferative activity against MDA-MB-231 compared to all combinations, with an IC_50_ value of 5.78 ± 0.06 mg/ml. With an IC_50_ value of 1.49 mg/ml, it was nearly as effective as Moringa-saffron against the T47-D cell line. However, its activity against EMT6/P was lower than that of the Moringa-saffron combination, at 5.78 ± 0.06 mg/ml.

The Moringa-lavender extract demonstrated activity on T47-D among the tested cell lines, with an IC_50_ value of 5.9 ± 2.41 mg/ml. In contrast, it exhibited a lower potential to inhibit the proliferation of EMT6/P and MDA-MB-231 cells, with an IC_50_ value greater than 6 mg/ml.

The Moringa-turmeric extract demonstrated anti-proliferative activity against the EMT6/P cell line, with an IC_50_ of 5.93 mg/ml; however, T47-D and MDA-MB-231 were unresponsive.

Uncombined Moringa displayed the lowest activity across all tested cell lines, with an IC_50_ exceeding 6 mg/ml.

Doxorubicin served as the positive control in the anti-proliferative trials. The cell lines T47-D, EMT6/P, MDA-MB231, and Vero displayed susceptibility to doxorubicin, with IC_50_ values of 0.87 ± 0.04, 4.74 ± 0.07, 7.35 ± 1.87, and over five µg/ml, respectively.

(**Table 3**) displays the IC_50_ values for doxorubicin and all extracts using the four cell lines.

**Table 3:** IC_50_ values of Moringa (*M. oleifera*) and its combinations’ extracts against T47-D, EMT6/P, MDA-MB-231, Vero cell lines.

Cell line	IC_50_ of Moringa (mg/ml)	IC_50_ of Moringa- saffron (mg/ml)	IC_50_ of Moringa- green tea (mg/ml)	IC_50_ of Moringa-lavender (mg/ml)	IC_50_ of Moringa-turmeric (mg/ml)	Doxorubicin (µg/ml)
T47-D	>6	1.14 ± 0.05	1.49 ± 0.1	5.9 ± 2.41	>6	0.87 ± 0.04
EMT-6/P	>6	1.65 ± 0.27	5.78 ± 0.06	>6	5.93 ± 0.21	4.74 ± 0.07
MDA-MB-231	>6	>6	5.78 ± 0.06	>6	>6	7.35 ± 1.87
Vero	>6	>6	>6	>6	>6	>5

### 3.5 Immune assays

#### 3.5.1 The effects of Moringa (*M. oleifera*) combinations’ extracts on spleen lymphocyte proliferation

**Figure 5:**
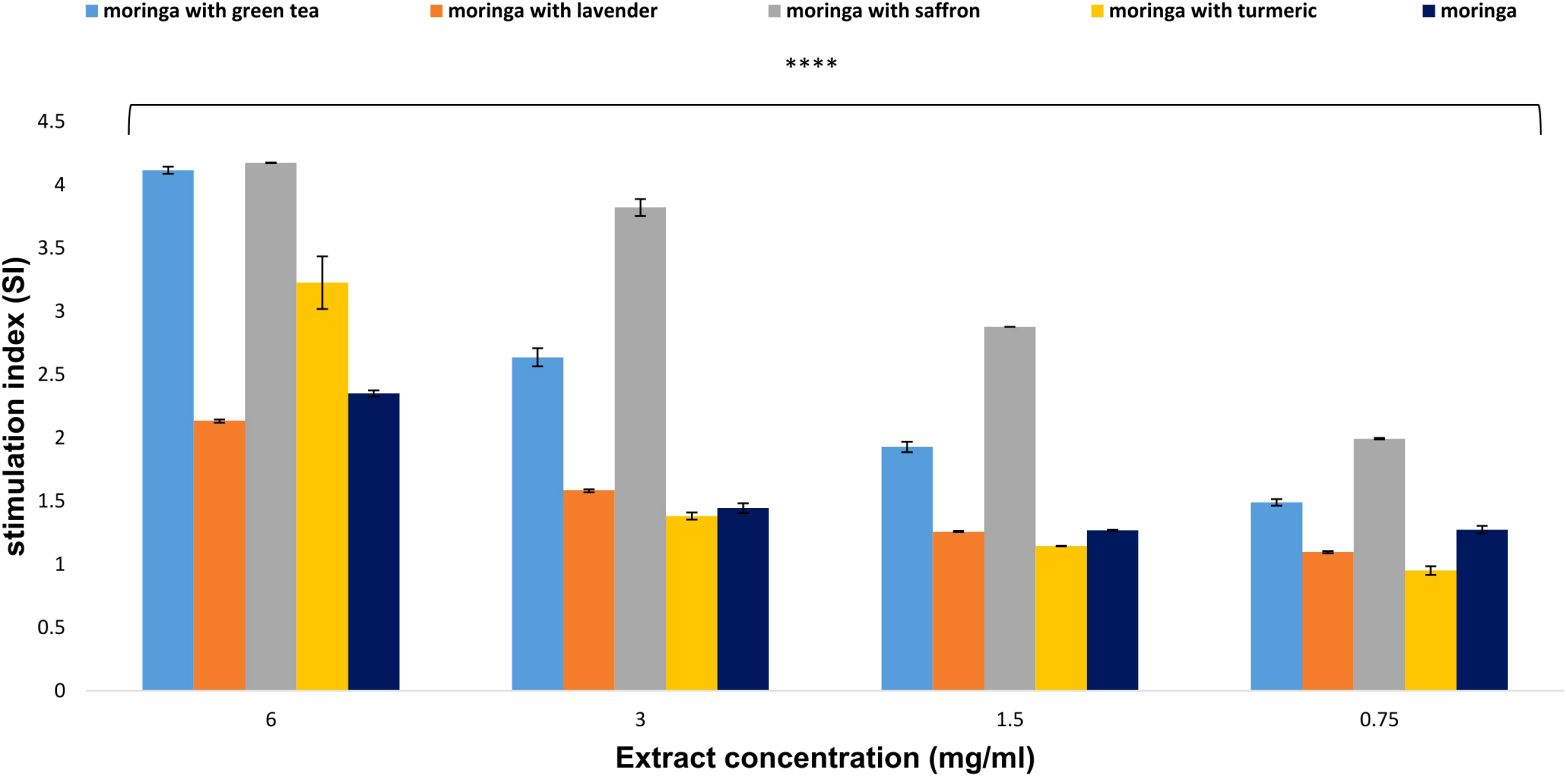
Effect of *Moringa oleifera* aqueous extract and its combinations on lymphocyte proliferation at various concentrations (6, 3, 1.5, and 0.75 mg/ml). Results are presented as mean ± SEM. Statistical comparisons were made against the negative control group (n = 3 independent experiments in technical triplicate). Statistical analysis was conducted using one-way ANOVA and Dunnett’s *post hoc* test. Significance is indicated as (****) *p*-value < 0.0001 significance level. Treatment concentrations were compared with the mean of the negative control group. A *p*-value < 0.05 was considered statistically significant. Error bars represent the SEM.

The results indicated that at a concentration of 6 mg/ml, the combination of Moringa with saffron and green tea exhibited the highest capacity to induce lymphocyte proliferation, demonstrating stimulation index values of 4.175 and 4.115, respectively. At the same dose, the combinations of Moringa-turmeric, Moringa-lavender, and uncombined Moringa showed lower stimulation indexes of 3.227, 2.136, and 2.351, respectively (**Figure 5**).

#### 3.5.2 The effects of Moringa (*M. oleifera*) combinations’ extracts on macrophage phagocytosis

**Figure 6:**
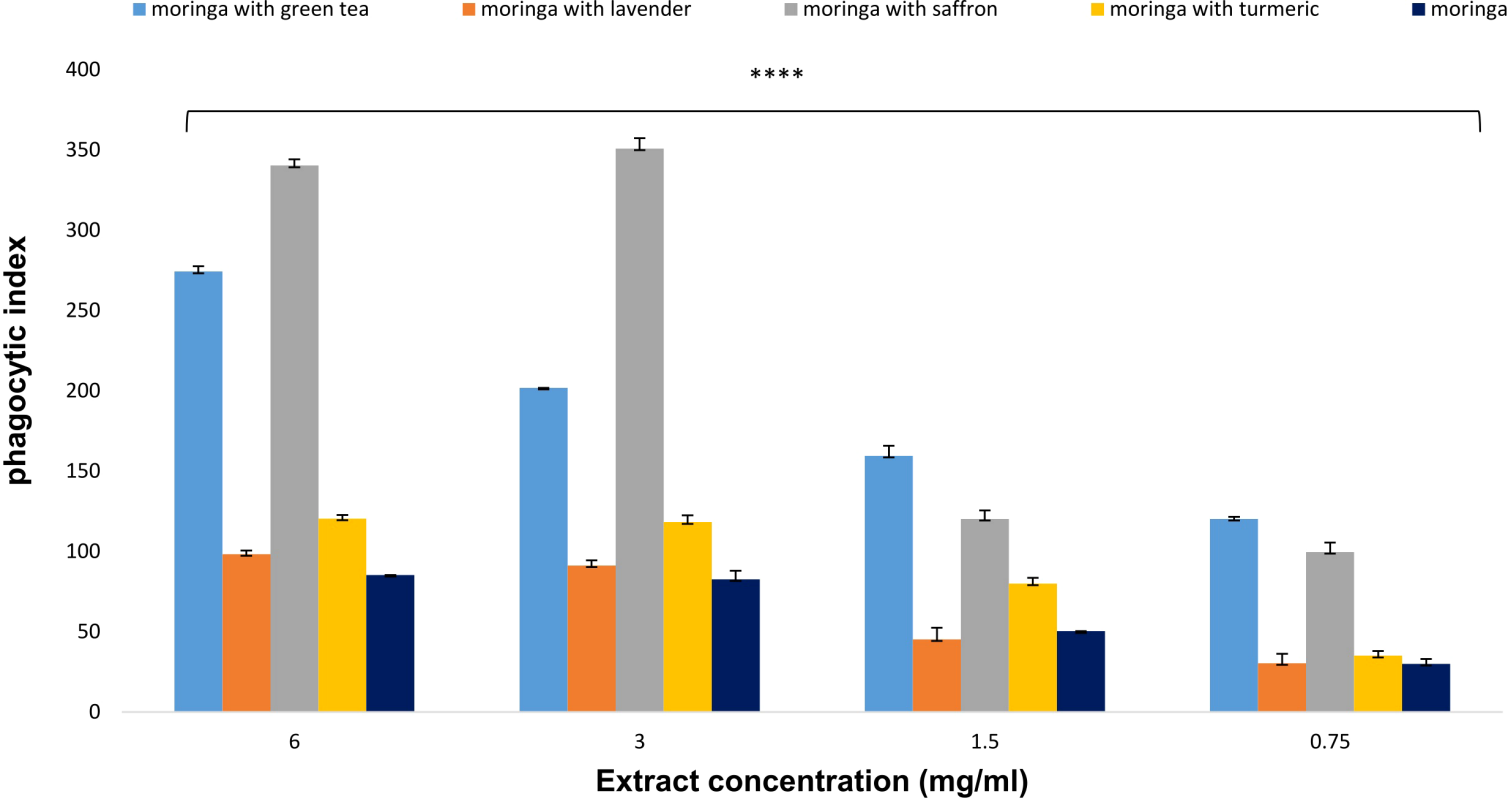
Effect of *Moringa oleifera* aqueous extract and its combinations on macrophage phagocytosis at various concentrations (6, 3, 1.5, and 0.75 mg/ml). Results are presented as mean ± SEM. Statistical analysis was conducted using one-way ANOVA and Dunnett’s *post hoc* test. Significance is indicated as (****) *p*-value < 0.0001 significance level. Treatment concentrations were compared with the mean of the negative control group. A *p*-value < 0.05 was considered statistically significant. Error bars represent the SEM.

The results indicated that at a concentration of 6 mg/ml, the extracts of Moringa-saffron and Moringa-green tea combinations were the most effective at inducing macrophage phagocytosis, showing close phagocytic index values of 340.28 and 274.29, respectively. However, at the same concentration, the combinations of Moringa-lavender, Moringa-turmeric, and uncombined Moringa displayed lower activities, with phagocytic indexes of 98.25, 120.325, and 85.250, respectively (**Figure 6**).

#### 3.5.3 The effects of Moringa (*M. oleifera*) combinations’ extracts on macrophage pinocytosis

**Figure 7:**
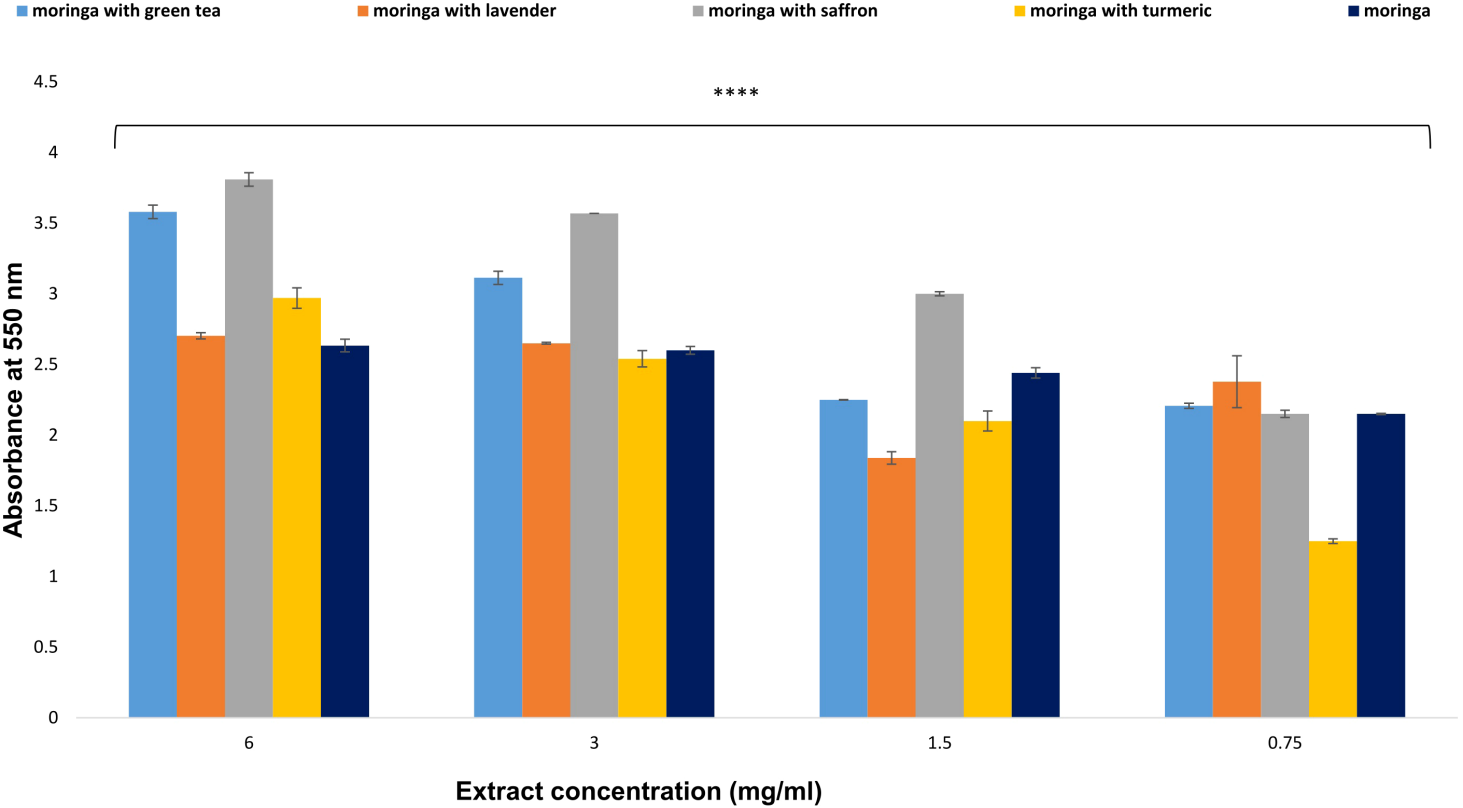
Effect of *Moringa oleifera* aqueous extract and its combinations on macrophage pinocytosis at various concentrations (6, 3, 1.5, and 0.75 mg/ml). Results are presented as mean ± SEM. Statistical analysis was conducted using one-way ANOVA and Dunnett’s *post hoc* test. Significance is indicated as (****) *p*-value < 0.0001 significance level. Treatment concentrations were compared with the mean of the negative control group. A *p*-value < 0.05 was considered statistically significant. Error bars represent the SEM.

The test aimed to determine the impact of Moringa extracts on the pinocytic activity of macrophages. The results indicated that the crude extracts enhanced and increased pinocytic activity at doses ranging from 0.75 to 6 mg/ml. Compared to the negative control’s optical density (0.313 ± 0.0067 nm), the Moringa-saffron and Moringa-green tea extracts exhibited the highest potential for inducing macrophage pinocytic activity, with absorbance values of 3.81 and 3.58, respectively. However, at the same concentration combinations, Moringa-lavender, Moringa-turmeric, and uncombined Moringa displayed lower activities, with absorbance values of 2.702, 2.97, and 2.63, respectively (**Figure 7**).

## 4 Discussion

Medicinal plants have played a crucial role in drug discovery, especially in oncology, where many chemotherapeutics are sourced from natural products (35). *Moringa oleifera*, widely cultivated in Southeast Asia, has various therapeutic properties, including antioxidant, immunomodulatory, and anticancer effects (36).

Moringa bark and leaf extracts have anticancer properties that could be utilized to develop novel medications for treating colorectal and breast cancers (37).

Previous studies confirm its activity against breast cancer cells, including the induction of apoptosis and a reduction in tumor volume in xenograft models (38–40).

Other investigations revealed that treatment with *M. oleifera* leaf extracts against MCF-7 breast cancer cells resulted in a significant decrease in cell viability, and up to 64.5% of solid tumors in MDA-MB-231 xenograft mice were suppressed in the third week following high-dose *M. oleifera* extract therapy (41).

This study utilized the maceration extraction method to create Moringa polyherbal infusions, imitating traditional preparation where herbal mixtures are steeped in hot water. This technique aimed to reflect real-world consumption habits and accurately represent the bioactive compounds in traditional practices, rather than concentrating solely on maximizing extraction yields through intensive methods.

**Figure 8:**
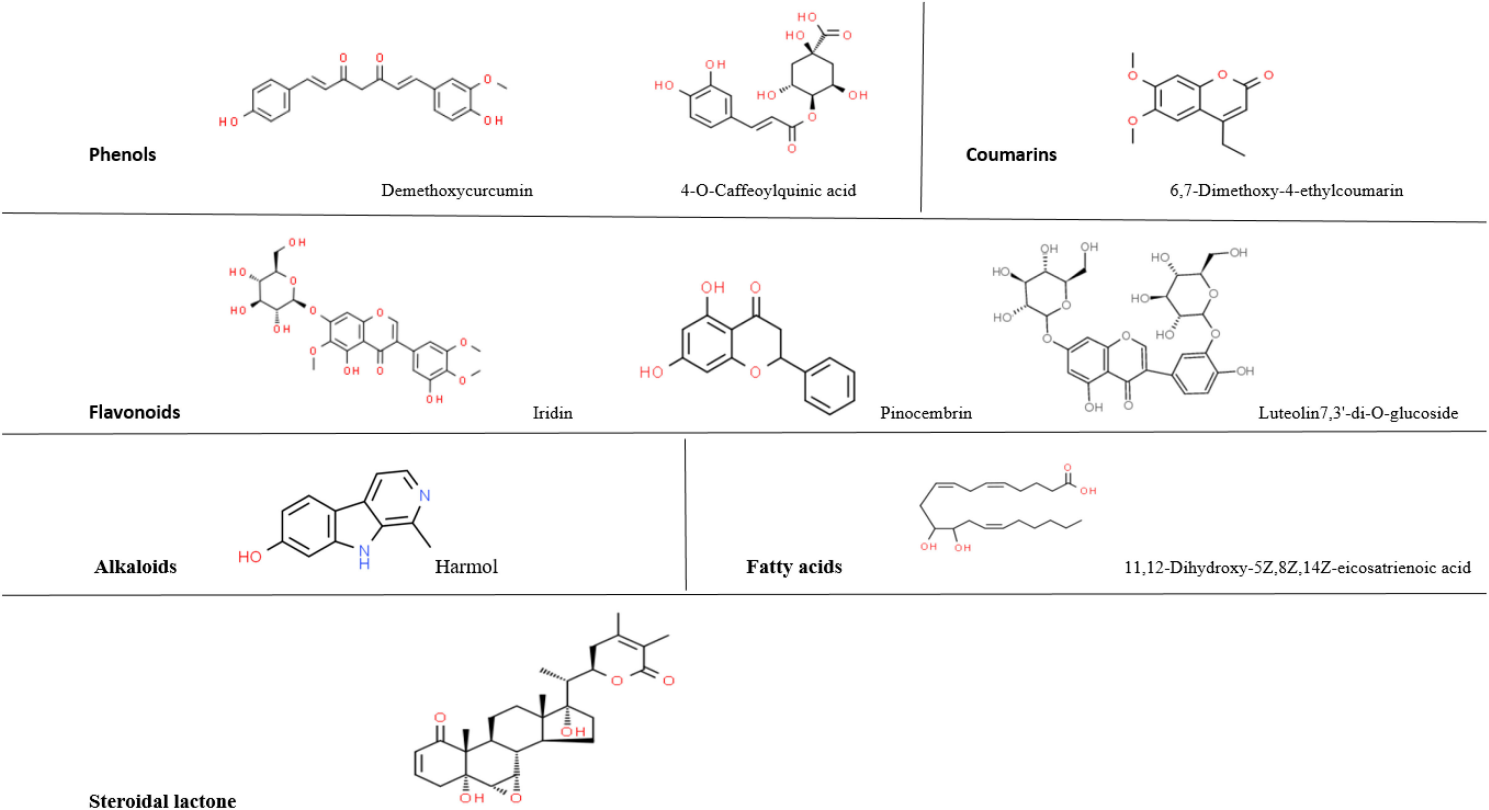
Structures of phytochemicals with anticancer and immunomodulatory activities found in Moringa-saffron combinations.

**Figure 9:**
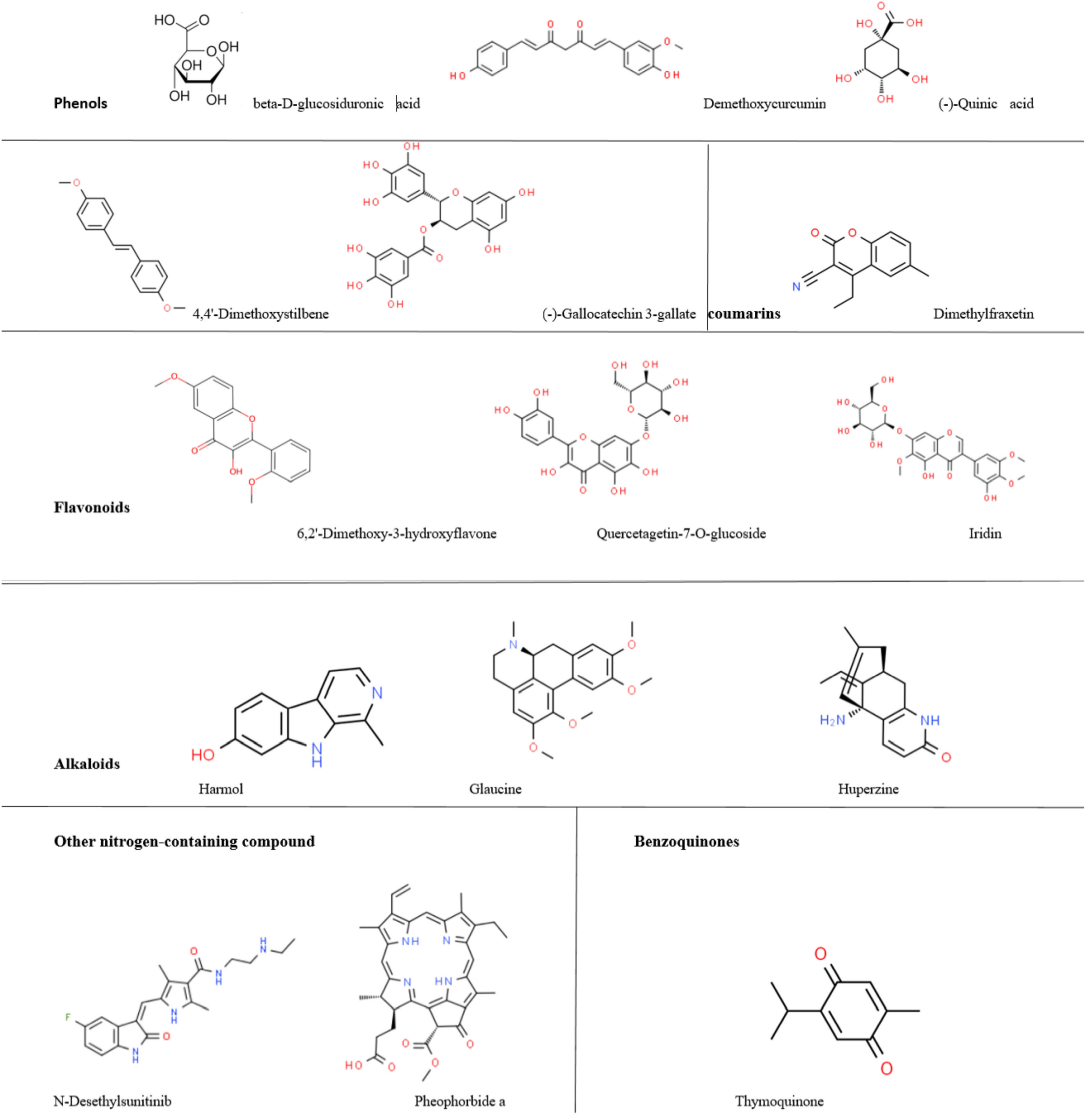
Structures of phytochemicals with anticancer and immunomodulatory activities found in Moringa-green tea combinations.

Afterward, *M. oleifera* leaf extract and its combinations—particularly with saffron and green tea—demonstrated potent anti-proliferative effects against T47-D, EMT6/P, and MDA-MB-231 breast cancer cell lines. Notably, the Moringa-saffron combination showed superior efficacy against T47-D and EMT6/P, while the Moringa-green tea combination was more effective against MDA-MB-231. These findings align with earlier reports suggesting that phytochemicals in these plants exert anti-proliferative activity through multiple pathways (**Figures 8**, **9**) (43–45).

A comparison of IC_50_ values with doxorubicin, which was utilized as a positive control in this assay, demonstrated that doxorubicin had greater potency with notable variations. These discrepancies could arise from the complex mixture of phytochemicals, potential antagonistic interactions, or lower concentrations of the active phytochemicals in the moringa combinations.

Phytochemical analysis using LC-MS/MS identified bioactive compounds such as demethoxycurcumin, 4-O-caffeoylquinic acid, gallocatechin 3-gallate, and dimethoxystilbene. These compounds influence apoptosis, immune modulation, and redox balance (46, 47). Flavonoids such as iridin, luteolin, and pinocembrin, found in the active combinations, inhibit tumor growth by targeting cell signaling, apoptosis, and angiogenesis (48).

The results revealed the presence of beta-D-glucosiduronic acid, a glucuronide conjugate of phenol that shares functional similarities with phenols, in the Moringa-green tea combination. Demethoxycurcumin, present in both combinations, is a curcuminoid phenol that modulates angiogenesis, metastasis, apoptosis, cell proliferation, and chemosensitivity (42).

Another study demonstrated that NCI-H460 human lung cancer cells underwent apoptosis when exposed to Demethoxycurcumin through a mitochondrial-dependent mechanism (43). Numerous studies have shown the immune-modulating effects of Demethoxycurcumin, which include reducing the production of pro-inflammatory cytokines and chemokines such as TNF-α, IL-1β, IL-6, and IL-8 while simultaneously increasing the levels of anti-inflammatory cytokines, notably IL-10 (44, 45).

Additionally, 4-O-Caffeoylquinic acid, a phenolic acid in the Moringa-saffron combination, has demonstrated notable antioxidant activity. A study revealed that this acid exhibited the highest levels of antioxidant effectiveness. Its significant role in predicting the antioxidant properties of the 12-month crude extracts was confirmed through the DPPH free radical-scavenging assay (46, 47). It also demonstrated considerable anti-inflammatory effects, indicating its ability to influence immune responses (48).

Investigations revealed that Dimethoxystilbene, present in the Moringa-green tea combination, preferentially stimulated autophagy and death in cancer cells while having minimal effects on non-cancerous cells (49). It also had antioxidant and immune-modulating properties, as evidenced by increased natural killer (NK) cell activity (50).

The phenol quinic acid influenced the tumor immune environment and exhibited a potential synergistic effect with anti-PD1 inhibition, which was investigated using a syngeneic colon tumor animal model (51).

It also regulates pro-inflammatory cytokines to inhibit cancer growth. It possesses immune-modulating properties by activating lymphocytes, critical components of the adaptive immune system (52).

A previous study showed that gallocatechin 3-gallate, a green tea polyphenol, influenced immunity by activating lymphocytes, which may have implications for treating breast cancer (53).

Some studies have explained flavonoids’ anti-carcinogenic properties, such as blocking the differentiation and growth of cancerous cells, reducing tumor angiogenesis, restricting vascular proliferation, and limiting metastasis and oxidative damage (54, 55).

However, flavonoids can support various immunomodulatory actions critical for controlling different types of cancer. Green tea catechin metabolites enhance the cytotoxicity of natural killer (NK) cells (56).

Iridin is a natural flavonoid detected in both Moringa-green tea and Moringa-saffron combinations. A current study indicated that Iridin therapy attenuated cell cycle arrest, reduced the proliferation of gastric cancer cells, and induced apoptosis (57).

Pinocembrin and luteolin are flavonoids detected in Moringa-saffron extract. Pinocembrin demonstrated anticancer activity in breast cancer models by activating apoptosis, stopping the cell cycle, and inhibiting the PI3K/AKT signaling pathway *in vivo* and *in vitro* (58).

According to a previous study (59), luteolin’s ability to inhibit tumor formation *in vivo*, prevent carcinogenesis in animal models, induce apoptosis, suppress angiogenesis, and enhance tumor cells’ vulnerability to the lethal effects of certain anticancer medications are proposed anticancer mechanisms.

In cancerous cells, coumarins exhibited antioxidant activity with low IC_50_ values (60, 61), significantly enhancing immunity (62).

Scientific research has demonstrated that they have anticancer effects based on their impact on immunological modulation, cell division, and growth. The majority of natural coumarins have usage restrictions due to their hepatotoxic effects; nevertheless, through molecular alterations, reasonably safe analogs with enhanced potency have been produced (63, 64).

The phytochemical classes in Moringa-green tea and Moringa-saffron combinations may account for their greater effects on antiproliferation and immunomodulatory functions than other combinations. However, these combinations exhibited some slight differences.

The presence of fatty acids and steroids in the Moringa-saffron combination, alongside their absence in Moringa-green tea, may explain their superiority over the Moringa-green tea combination against T47-D and EMT6/P cell lines. These results align with previous studies indicating that the anti-proliferative activity occurs through a variety of mechanisms, which include effects on signal transduction, transcription factor activity, gene expression, and estrogen metabolism; alterations in the generation of free radicals and reactive oxygen species; suppression of neoplastic transformation; inhibition of cell growth; and enhanced apoptosis in breast cancer cell lines (65, 66).

This may also be linked to the superiority observed among all combinations in the immune tests conducted in this investigation, a notion also suggested by various studies (67, 68).

The natural steroidal lactone, Withanone, may also be linked to the highest efficiency in this combination. Through the production of ROS signaling, Withanone selectively kills cancer cells, making it a suitable reagent for ROS-mediated cancer treatment (69). This could also be related to its potential inhibition of survivin, an inhibitor of apoptosis that is highly expressed in many cancer cell lines (70). Additionally, it modulated the immune response in Balb/c mice in a previous study (71).

In contrast, LC-MS/MS demonstrated the presence of many alkaloids in the Moringa-green tea combination, including Harmol, Glaucine, and Huperzine, which could be the reason behind the superior activity of this combination on the MDA-MB-231 cell line. A previous study suggested that the β-carboline alkaloid Harmol activates caspase-8 to induce apoptosis. This results in the autophagy and eventual death of human NSCLC A549 cells (72).

By suppressing NF-κB activation, glaucine decreases the production of the MMP-9 gene, thereby limiting the migration and invasion of breast cancer cells (73) and modulating the immune response, primarily by reducing pro-inflammatory cytokine production and influencing intracellular signaling pathways in immune cells (74, 75).

N-Desethylsunitinib and Pheophorbide A, nitrogen-containing compounds, were detected in the Moringa-green tea combination. A study suggested that these agents effectively treat lymphomas, breast cancer, lung cancer, and leukemia (76).

Thymoquinone, a benzoquinone found in the Moringa-green tea combination, could be responsible for the anti-proliferative and cytotoxic activity on the MDA-MB-231 cell line (77, 78).

A previous study showed that Pheophorbide A can activate murine RAW 264.7 macrophages without light stimulation. This activation resulted in higher production of nitric oxide (NO) and pro-inflammatory cytokines like TNF-α and IL-6, suggesting Pa’s ability to boost innate immune responses without relying on photodynamic therapy (79). Another study revealed that Pa-mediated photodynamic therapy (PDT) increases the expression of HLA class I molecules, which are essential for antigen presentation. This enhancement supports the activation of cytotoxic T lymphocytes, thereby advancing adaptive immune responses against tumor cells (80).

Our findings correlate with the reports from the previously mentioned investigations on the numerous fascinating phytochemicals discovered in *M. oleifera* (Moringa) aqueous leaf extract combinations of green tea or saffron. These mechanistic insights suggest that the observed bioactivity results from the concerted action of multiple phytochemicals targeting diverse cancer-related pathways, including oxidative stress regulation, immune modulation, apoptosis induction, and inhibition of cell migration and proliferation.

Given these encouraging *in vitro* results, future studies should validate the therapeutic efficacy of these combinations using *in vivo* tumor models. Furthermore, isolating and testing individual compounds will help clarify their specific contributions and potential pharmacological interactions, laying the groundwork for developing innovative phytochemical-based therapeutics.

## 5 Conclusion

Combining natural products with proven anticancer properties and synergistic interactions enhances the search for potential molecular targets in cancer cells. According to this study’s findings, aqueous leaf extract from *M. oleifera* was able to halt the proliferation of breast cancer cells both independently and in conjunction with other plants. The most effective combinations were with saffron and green tea, which exhibited low IC_50_ values and more significant immunomodulatory actions. Immunomodulatory experiments with *M. oleifera* and its combinations indicated that the plant effectively enhanced macrophage phagocytosis, pinocytosis, and lymphocyte proliferation. Furthermore, the phytochemical examination revealed the presence of numerous bioactive compounds possessing known anti-proliferative, antioxidant, and immunomodulatory properties, including phenols, flavonoids, coumarins, alkaloids, benzoquinones, and fatty acids in moringa-green tea and moringa-saffron combinations.

To our knowledge, this study is the first to investigate the anticancer and immunomodulatory effects of moringa combinations. These results establish a strong foundation for future research. However, this study has limitations, particularly identifying specific molecular targets and signaling pathways that moringa-saffron and green tea combinations affect. Future research should aim to isolate and characterize the pure active components of these combinations and clarify the molecular mechanisms involved, including the expression of cytokines such as INF-γ and IL-6/7/8. Understanding these pathways will be essential for applying moringa-saffron and green tea combinations in cancer treatment.

Additionally, future *in vivo* studies are essential for a comprehensive understanding of these moringa combinations’ bioavailability, metabolism, and potential toxicity.

## Data Availability

The original contributions presented in the study are included in the article/supplementary material. Further inquiries can be directed to the corresponding author.

## References

[1] Bai J Barandouzi ZA Rowcliffe C Meador R Tsementzi D Bruner DW. Gut microbiome and its associations with acute and chronic gastrointestinal toxicities in cancer patients with pelvic radiation therapy: a systematic review. Front Oncol. (2021) 11:745262. doi: 10.3389/fonc.2021.745262 34938654 PMC8685326

[2] Thun MJ DeLancey JO Center MM Jemal A Ward EM. The global burden of cancer: priorities for prevention. Carcinogenesis. (2010) 31:100–10. doi: 10.1093/carcin/bgp263 PMC2802672 19934210

[3] Rohr JR Barrett CB Civitello DJ Craft ME Delius B DeLeo GA. Emerging human infectious diseases and the links to global food production. Nat sustainabil. (2019) 2:445–56. doi: 10.1038/s41893-019-0293-3 PMC7091874 32219187

[4] Ferlay J Colombet M Soerjomataram I Parkin DM Piñeros M Znaor A. Cancer statistics for the year 2020: An overview. Int J cancer. (2021) 149:778–89. doi: 10.1002/ijc.v149.4 33818764

[5] Pearce A Haas M Viney R Pearson S-A Haywood P Brown C. Incidence and severity of self-reported chemotherapy side effects in routine care: A prospective cohort study. PloS One. (2017) 12:e0184360. doi: 10.1371/journal.pone.0184360 29016607 PMC5634543

[6] Parihar A Puranik N Nadda AK Kumar V Lee KW Kumar R. Phytochemicals for breast cancer therapeutic intervention: exploratory in silico molecular docking study. Medinformatics. (2024) 1–15. doi: 10.47852/bonviewMEDIN42023059

[7] Parihar A Ahmed SS Sharma P Choudhary NK Akter F Ali MA. Plant-based bioactive molecules for targeting of endoribonuclease using steered molecular dynamic simulation approach: a highly conserved therapeutic target against variants of SARS-CoV-2. Mol Simul. (2023) 49:1267–79. doi: 10.1080/08927022.2022.2113811

[8] Parihar A Sonia ZF Akter F Ali MA Hakim FT Hossain MS. Phytochemicals-based targeting RdRp and main protease of SARS-CoV-2 using docking and steered molecular dynamic simulation: A promising therapeutic approach for Tackling COVID-19. Comput Biol Med. (2022) 145:105468. doi: 10.1016/j.compbiomed.2022.105468 35390745 PMC8964014

[9] Gonzales GF Valerio LG. Medicinal plants from Peru: a review of plants as potential agents against cancer. Anti-Cancer Agents Med Chemistry-Anti-Cancer Agents). (2006) 6:429–44. doi: 10.2174/187152006778226486 17017852

[10] Jamshidi-Kia F Lorigooini Z Amini-Khoei H. Medicinal plants: Past history and future perspective. J herbmed Pharmacol. (2017) 7:1–7. doi: 10.15171/jhp.2018.01

[11] Sauter ER. Cancer prevention and treatment using combination therapy with natural compounds. Expert Rev Clin Pharmacol. (2020) 13:265–85. doi: 10.1080/17512433.2020.1738218 32154753

[12] Dillman RO. Cancer immunotherapy. Cancer biother radiopharma. (2011) 26:1–64. doi: 10.1089/cbr.2010.0902 21355777

[13] Huang Q Pan X Zhu W Zhao W Xu H Hu K. Natural products for the immunotherapy of glioma. Nutrients. (2023) 15:2795. doi: 10.3390/nu15122795 37375698 PMC10302066

[14] Dhakad AK Ikram M Sharma S Khan S Pandey VV Singh A. Biological, nutritional, and therapeutic significance of Moringa oleifera Lam. Phytother Res. (2019) 33:2870–903. doi: 10.1002/ptr.v33.11 31453658

[15] Anwar F Ashraf M Bhanger MI. Interprovenance variation in the composition of Moringa oleifera oilseeds from Pakistan. J Am Oil Chemists’ Soc. (2005) 82:45–51. doi: 10.1007/s11746-005-1041-1

[16] Farooq F Rai M Tiwari A Khan AA Farooq S. Medicinal properties of Moringa oleifera: An overview of promising healer. J Med Plants Res. (2012) 6:4368–74. doi: 10.5897/JMPR12.279

[17] Pal D Thakur S Sahu T Hait M. Exploring the anticancer potential and phytochemistry of Moringa oleifera: a multi-targeted medicinal herb from nature. ES Food Agroforest. (2023) 14:982. doi: 10.30919/esfaf982

[18] Greenwell M Rahman P. Medicinal plants: their use in anticancer treatment. Int J Pharm Sci Res. (2015) 6:4103. doi: 10.13040/IJPSR.0975-8232.6(10).4103-12 26594645 PMC4650206

[19] Mansoori B Mohammadi A Davudian S Shirjang S Baradaran B. The different mechanisms of cancer drug resistance: a brief review. Adv Pharm bullet. (2017) 7:339. doi: 10.15171/apb.2017.041 PMC5651054 29071215

[20] Wang H Oo Khor T Shu L Su Z-Y Fuentes F Lee J-H. Plants vs. cancer: a review on natural phytochemicals in preventing and treating cancers and their druggability. Anticancer Agents Med Chem (Formerly Curr Med Chemistry-Anti-Cancer Agents). (2012) 12:1281–305. doi: 10.2174/187152012803833026 PMC4017674 22583408

[21] Lambrianidou A Koutsougianni F Papapostolou I Dimas K. Recent advances on the anticancer properties of saffron (Crocus sativus L.) and its major constituents. Molecules. (2020) 26:86. doi: 10.3390/molecules26010086 33375488 PMC7794691

[22] Samarghandian S Borji A. Anti-carcinogenic effect of saffron (Crocus sativus L.) and its ingredients. Pharmacogn Res. (2014) 6:99. doi: 10.4103/0974-8490.128963 PMC3996758 24761112

[23] Chermahini SH Majid FAA Sarmidi MR Taghizadeh E Salehnezhad S. Impact of saffron as an anticancer and anti-tumor herb. Afr J Pharm Pharmacol. (2010) 4:834–40. Available online at: http://www.academicjournals.org/ajpp.

[24] Fahmy MA Farghaly AA Hassan EE Hassan EM Hassan ZM Mahmoud K. Evaluation of the anticancer/anti-mutagenic efficiency of Lavandula officinalis essential oil. Asian Pac J Cancer prevent: APJCP. (2022) 23:1215. doi: 10.31557/APJCP.2022.23.4.1215 PMC9375616 35485678

[25] Mousavi SH Afshari JT Brook A. Study of cytotoxic effects of saffron in MCF-7 cells: cytotoxicity of saffron. Iran J Pharm Sci. (2008) 4:261–8. doi: 10.22037/ijps.v4.41052

[26] Hashemi SA Karami M Bathaie SZ. Saffron carotenoids change the superoxide dismutase activity in breast cancer: *In vitro*, *in vivo* and in silico studies. Int J Biol macromol. (2020) 158:845–53. doi: 10.1016/j.ijbiomac.2020.04.063 32360463

[27] Zhao Y Chen R Wang Y Qing C Wang W Yang Y. *In vitro* and *in vivo* efficacy studies of lavender angustifolia essential oil and its active constituents on the proliferation of human prostate cancer. Integr Cancer ther. (2017) 16:215–26. doi: 10.1177/1534735416645408 PMC5739122 27151584

[28] Boukhatem MN Sudha T Darwish NH Chader H Belkadi A Rajabi M. A new eucalyptol-rich lavender (Lavandula stoechas L.) essential oil: Emerging potential for therapy against inflammation and cancer. Molecules. (2020) 25:3671. doi: 10.3390/molecules25163671 32806608 PMC7463424

[29] Wu AH Butler LM. Green tea and breast cancer. Mol Nutr Food Res. (2011) 55:921–30. doi: 10.1002/mnfr.201100006 PMC4196858 21538855

[30] Nakachi K Suemasu K Suga K Takeo T Imai K Higashi Y. Influence of drinking green tea on breast cancer Malignancy among Japanese patients. Japan J Cancer Res. (1998) 89:254–61. doi: 10.1111/j.1349-7006.1998.tb00556.x PMC5921805 9600118

[31] Wright L E Frye J B Gorti B N Timmermann B L Funk J. Bioactivity of turmeric-derived curcuminoids and related metabolites in breast cancer. Curr Pharm design. (2013) 19:6218–25. doi: 10.2174/1381612811319340013 PMC3883055 23448448

[32] Fabianowska-Majewska K Kaufman-Szymczyk A Szymanska-Kolba A Jakubik J Majewski G Lubecka K. Curcumin from turmeric rhizome: a potential modulator of DNA methylation machinery in breast cancer inhibition. Nutrients. (2021) 13:332. doi: 10.3390/nu13020332 33498667 PMC7910847

[33] Al Kury LT Taha Z Mahmod AI Talib WH. Xanthium spinosum L. extracts inhibit breast cancer in mice by apoptosis induction and immune system modulation. Pharmaceuticals. (2022) 15:1504. doi: 10.3390/ph15121504 36558955 PMC9784301

[34] Boothapandi M Ramanibai R. Immunomodulatory activity of Indigofera tinctoria leaf extract on *in vitro* macrophage responses and lymphocyte proliferation. Int J Pharm Pharm Sci. (2016) 8:58–63. Available online at: https://journals.innovareacademics.in/index.php/ijpps/article/view/9932/6177.

[35] Solowey E Lichtenstein M Sallon S Paavilainen H Solowey E Lorberboum-Galski H. Evaluating medicinal plants for anticancer activity. Sci World J. (2014) 2014:721402. doi: 10.1155/2014/721402 PMC4248331 25478599

[36] Abdull Razis AF Ibrahim MD Kntayya SB. Health benefits of Moringa oleifera. Asian pac J Cancer Prev. (2014) 15:8571–6. doi: 10.7314/APJCP.2014.15.20.8571 25374169

[37] Al-Asmari AK Albalawi SM Athar MT Khan AQ Al-Shahrani H Islam M. Moringa oleifera as an anticancer agent against breast and colorectal cancer cell lines. PloS One. (2015) 10:e0135814. doi: 10.1371/journal.pone.0135814 26288313 PMC4545797

[38] Hossain N Mirghani M Raus RB. Optimization of Moringa oleifera leaf extraction and investigation of anti breast cancer activity with the leaf extract. Eng Int. (2015) 3:97. doi: 10.18034/ei.v3i2.194

[39] Gaffar S Apriani R Herlina T Gaffar S Apriani R Herlina T. n-Hexane fraction of Moringa oleifera Lam. leaves induces apoptosis and cell cycle arrest on T47D breast cancer cell line. J Pharm Pharmacogn Res. (2019) 7:173–83. doi: 10.56499/jppres18.477_7.3.173

[40] Szlachetka K Kut P Stępień A. Cytotoxic and anticancer activity of Moringa oleifera. Eur J Clin Exp Med. (2020) 18(3):214–20. doi: 10.15584/ejcem.2020.3.9

[41] Lim WF Mohamad Yusof MI Teh LK Salleh MZ. Significant decreased CaN, VEGF, SLC39A6 and SFRP1 expressions in MDA-MB-231 xenograft breast tumor mice treated with Moringa oleifera leaves and seed residue (MOLSr) extracts. Nutrients. (2020) 12:2993. doi: 10.3390/nu12102993 33007803 PMC7601446

[42] Hatamipour M Ramezani M Tabassi SAS Johnston TP Ramezani M Sahebkar A. Demethoxycurcumin: A naturally occurring curcumin analogue with anti-tumor properties. J Cell Physiol. (2018) 233:9247–60. doi: 10.1002/jcp.v233.12 30076727

[43] Ko Y-C Lien J-C Liu H-C Hsu S-C Ji B-C Yang M-D. Demethoxycurcumin induces the apoptosis of human lung cancer NCI-H460 cells through the mitochondrial-dependent pathway. Oncol Rep. (2015) 33:2429–37. doi: 10.3892/or.2015.3865 25813094

[44] Boroumand N Samarghandian S Hashemy SI. Immunomodulatory, anti-inflammatory, and antioxidant effects of curcumin. J Herbmed Pharmacol. (2018) 7:211–9. doi: 10.15171/jhp.2018.33

[45] Kahkhaie KR Mirhosseini A Aliabadi A Mohammadi A Mousavi MJ Haftcheshmeh SM. Curcumin: a modulator of inflammatory signaling pathways in the immune system. Inflammopharmacology. (2019) 27:885–900. doi: 10.1007/s10787-019-00607-3 31140036

[46] Ganzon JG Chen L-G Wang C-C. 4-O-Caffeoylquinic acid as an antioxidant marker for mulberry leaves rich in phenolic compounds. J Food Drug anal. (2018) 26:985–93. doi: 10.1016/j.jfda.2017.11.011 PMC9303035 29976416

[47] Wong SK Lim YY Ling SK Chan EWC. Caffeoylquinic acids in leaves of selected Apocynaceae species: Their isolation and content. Pharmacogn Res. (2014) 6:67. doi: 10.4103/0974-8490.122921 PMC3897013 24497746

[48] Wu T-Y Chen C-C Lin J-Y. Anti-inflammatory *in vitro* activities of eleven selected caffeic acid derivatives based on a combination of pro–/anti-inflammatory cytokine secretions and principal component analysis–A comprehensive evaluation. Food Chem. (2024) 458:140201. doi: 10.1016/j.foodchem.2024.140201 38943957

[49] Yousuf M Jinka S Adhikari SS Banerjee R. Methoxy-enriched cationic stilbenes as anticancer therapeutics. Bioorg Chem. (2020) 98:103719. doi: 10.1016/j.bioorg.2020.103719 32171988

[50] Espinoza JL An DT Trung LQ Yamada K Nakao S Takami A. Stilbene derivatives from melinjo extract have antioxidant and immune modulatory effects in healthy individuals. Integr Mol Med. (2015) 2:405–13. doi: 10.15761/IMM.1000177

[51] Lin H Wu Y Chen J Huang S Wang Y. (–)-4-O-(4-O-β-D-glucopyranosylcaffeoyl) quinic acid inhibits the function of myeloid-derived suppressor cells to enhance the efficacy of anti-PD1 against colon cancer. Pharm Res. (2018) 35:1–9. doi: 10.1007/s11095-018-2459-5 30062658

[52] Baraya YS Wong KK Yaacob NS. The immunomodulatory potential of selected bioactive plant-based compounds in breast cancer: a review. Anticancer Agents Med Chem (Formerly Curr Med Chemistry-Anti-Cancer Agents). (2017) 17:770–83. doi: 10.2174/1871520616666160817111242 27539316

[53] Jang J-Y Lee J-K Jeon Y-K Kim C-W. Exosome derived from epigallocatechin gallate treated breast cancer cells suppresses tumor growth by inhibiting tumor-associated macrophage infiltration and M2 polarization. BMC cancer. (2013) 13:1–12. doi: 10.1186/1471-2407-13-421 24044575 PMC3848851

[54] Pietta P-G. Flavonoids as antioxidants. J Natural prod. (2000) 63:1035–42. doi: 10.1021/np9904509 10924197

[55] Nguyen NH Nguyen TT Ma PC Ta QTH Duong T-H Vo VG. Potential antimicrobial and anticancer activities of an ethanol extract from bouea macrophylla. Molecules. (2020) 25:1996. doi: 10.3390/molecules25081996 32344601 PMC7221966

[56] Wang S Li Z Ma Y Liu Y Lin C-C Li S. Immunomodulatory effects of green tea polyphenols. Molecules. (2021) 26:3755. doi: 10.3390/molecules26123755 34203004 PMC8234133

[57] Bhosale P-B Vetrivel P Ha S-E Kim H-H Heo J-D Won C-K. Iridin induces G2/M phase cell cycle arrest and extrinsic apoptotic cell death through PI3K/AKT signaling pathway in AGS gastric cancer cells. Molecules. (2021) 26:2802. doi: 10.3390/molecules26092802 34068568 PMC8126061

[58] Elbatreek MH Mahdi I Ouchari W Mahmoud MF Sobeh M. Current advances on the therapeutic potential of pinocembrin: an updated review. Biomed Pharmacother. (2023) 157:114032. doi: 10.1016/j.biopha.2022.114032 36481404

[59] López-Lázaro M. Distribution and biological activities of the flavonoid luteolin. Mini Rev med Chem. (2009) 9:31–59. doi: 10.2174/138955709787001712 19149659

[60] Alagesan V Ramalingam S Kim M Venugopal S. Antioxidant activity guided isolation of a coumarin compound from Ipomoea pes-caprea (Convolvulaceae) leaves acetone extract and its biological and molecular docking studies. Eur J Integr Med. (2019) 32:100984. doi: 10.1016/j.eujim.2019.100984

[61] Kostova I. Synthetic and natural coumarins as cytotoxic agents. Curr Med Chemistry-Anti-Cancer Agents. (2005) 5:29–46. doi: 10.2174/1568011053352550 15720259

[62] Khasamwala RH Ranjani S Nivetha SS Hemalatha S. COVID-19: An in silico analysis on potential therapeutic uses of Trikadu as immune system boosters. Appl Biochem Biotechnol. (2022) 194:291–301. doi: 10.1007/s12010-021-03793-5 PMC8731194 34988845

[63] Zhang L Xu Z. Coumarin-containing hybrids and their anticancer activities. Eur J Med Chem. (2019) 181:111587. doi: 10.1016/j.ejmech.2019.111587 31404864

[64] Rawat A Reddy AVB. Recent advances on anticancer activity of coumarin derivatives. Eur J Med Chem Rep. (2022) 5:100038. doi: 10.1016/j.ejmcr.2022.100038

[65] Pacheco BS Dos Santos MAZ Schultze E Martins RM Lund RG Seixas FK. Cytotoxic activity of fatty acids from Antarctic macroalgae on the growth of human breast cancer cells. Front bioeng Biotechnol. (2018) 6:185. doi: 10.3389/fbioe.2018.00185 30560124 PMC6286972

[66] Wannous R Bon E Mahéo K Goupille C Chamouton J Bougnoux P. PPARβ mRNA expression, reduced by n– 3 PUFA diet in mammary tumor, controls breast cancer cell growth. Biochim Biophys Acta (BBA)-Mol Cell Biol Lipids. (2013) 1831:1618–25. doi: 10.1016/j.bbalip.2013.07.010 23906790

[67] Stulnig TM. Immunomodulation by polyunsaturated fatty acids: mechanisms and effects. Int Arch Allergy Immunol. (2003) 132:310–21. doi: 10.1159/000074898 14707462

[68] Miles EA Calder PC. Modulation of immune function by dietary fatty acids. Proc Nutr Soc. (1998) 57:277–92. doi: 10.1079/PNS19980042 9656331

[69] Widodo N Priyandoko D Shah N Wadhwa R Kaul SC. Selective killing of cancer cells by Ashwagandha leaf extract and its component Withanone involves ROS signaling. PloS One. (2010) 5:e13536. doi: 10.1371/journal.pone.0013536 20975835 PMC2958829

[70] Wadegaonkar VP Wadegaonkar PA. Withanone as an inhibitor of survivin: A potential drug candidate for cancer therapy. J Biotechnol. (2013) 168:229–33. doi: 10.1016/j.jbiotec.2013.08.028 23994265

[71] Kushwaha S Roy S Maity R Mallick A Soni VK Singh PK. Chemotypical variations in Withania somnifera lead to differentially modulated immune response in BALB/c mice. Vaccine. (2012) 30:1083–93. doi: 10.1016/j.vaccine.2011.12.031 22182427

[72] Abe A Yamada H. Harmol induces apoptosis by caspase-8 activation independently on Fas/Fas ligand interaction in human lung carcinoma H596 cells. Anticancer Drugs. (2009) 20:373–81. doi: 10.1097/CAD.0b013e32832a2dd9 19318910

[73] Kang H Jang S-W Pak JH Shim S. Glaucine inhibits breast cancer cell migration and invasion by inhibiting MMP-9 gene expression through the suppression of NF-κB activation. Mol Cell Biochem. (2015) 403:85–94. doi: 10.1007/s11010-015-2339-9 25670016 PMC4383818

[74] Remichkova M Dimitrova P Philipov S Ivanovska N. Toll-like receptor-mediated anti-inflammatory action of glaucine and oxoglaucine. Fitoterapia. (2009) 80:411–4. doi: 10.1016/j.fitote.2009.05.016 19481591

[75] Cortijo J Villagrasa V Pons R Berto L Martí-Cabrera M Martinez-Losa M. Bronchodilator and anti-inflammatory activities of glaucine: *In vitro* studies in human airway smooth muscle and polymorphonuclear leukocytes. Br J Pharmacol. (1999) 127:1641–51. doi: 10.1038/sj.bjp.0702702 PMC1566148 10455321

[76] Lang DK Kaur R Arora R Saini B Arora S. Nitrogen-containing heterocycles as anticancer agents: An overview. Anticancer Agents Med Chem (Formerly Curr Med Chemistry-Anti-Cancer Agents). (2020) 20:2150–68. doi: 10.2174/1871520620666200705214917 32628593

[77] Adinew GM Messeha SS Taka E Badisa RB Antonie LM Soliman KF. Thymoquinone alterations of the apoptotic gene expressions and cell cycle arrest in genetically distinct triple-negative breast cancer cells. Nutrients. (2022) 14:2120. doi: 10.3390/nu14102120 35631261 PMC9144154

[78] Mostofa A Hossain MK Basak D Bin Sayeed MS. Thymoquinone as a potential adjuvant therapy for cancer treatment: evidence from preclinical studies. Front Pharmacol. (2017) 8:295. doi: 10.3389/fphar.2017.00295 28659794 PMC5466966

[79] Bui-Xuan N-H Tang PM-K Wong C-K Chan JY-W Cheung KKY Jiang JL. Pheophorbide a: A photosensitizer with immunostimulating activities on mouse macrophage RAW 264.7 cells in the absence of irradiation. Cell Immunol. (2011) 269:60–7. doi: 10.1016/j.cellimm.2011.02.010 21481339

[80] Tang PM-K Bui-Xuan N-H Wong C-K Fong W-P Fung K-P. Pheophorbide a-mediated photodynamic therapy triggers HLA class I-restricted antigen presentation in human hepatocellular carcinoma. Trans Oncol. (2010) 3:114–22. doi: 10.1593/tlo.09262 PMC2847319 20360936

